# Constitutively active Notch1 converts cranial neural crest-derived frontonasal mesenchyme to perivascular cells *in vivo*

**DOI:** 10.1242/bio.023887

**Published:** 2017-02-09

**Authors:** Sophie R. Miller, Surangi N. Perera, Clare V. H. Baker

**Affiliations:** Department of Physiology, Development and Neuroscience, University of Cambridge, Anatomy Building, Downing Street, Cambridge CB2 3DY, UK

**Keywords:** Notch, Pericyte, Neural crest, Frontonasal mesenchyme, Olfactory ensheathing cells, Chick embryo

## Abstract

Perivascular/mural cells originate from either the mesoderm or the cranial neural crest. Regardless of their origin, Notch signalling is necessary for their formation. Furthermore, in both chicken and mouse, constitutive Notch1 activation (via expression of the Notch1 intracellular domain) is sufficient *in vivo* to convert trunk mesoderm-derived somite cells to perivascular cells, at the expense of skeletal muscle. In experiments originally designed to investigate the effect of premature Notch1 activation on the development of neural crest-derived olfactory ensheathing glial cells (OECs), we used *in ovo* electroporation to insert a tetracycline-inducible *NotchΔE* construct (encoding a constitutively active mutant of mouse Notch1) into the genome of chicken cranial neural crest cell precursors, and activated *NotchΔE* expression by doxycycline injection at embryonic day 4. *NotchΔE*-targeted cells formed perivascular cells within the frontonasal mesenchyme, and expressed a perivascular marker on the olfactory nerve. Hence, constitutively activating Notch1 is sufficient *in vivo* to drive not only somite cells, but also neural crest-derived frontonasal mesenchyme and perhaps developing OECs, to a perivascular cell fate. These results also highlight the plasticity of neural crest-derived mesenchyme and glia.

## Abstract (precis)

**Summary:** Sustained Notch1 activation is sufficient to drive cranial neural crest-derived frontonasal mesenchyme to adopt a perivascular (mural) cell fate in developing chick embryos.

## Dedication

This paper is dedicated to the memory of Dr Sophie R. Miller, who passed away in December 2016.

## INTRODUCTION

Perivascular (mural) cells – pericytes and vascular smooth muscle cells – form the periendothelial (outer) wall of blood vessels: mature pericytes are embedded within the basement membrane of the endothelial cells in microvessels (capillaries, terminal arterioles, postcapillary venules), while vascular smooth muscle cells are found in multiple layers around larger vessels (reviewed by Armulik et al., 2011; Majesky et al., 2011). Perivascular cells in the trunk, and many in the head, originate from mesoderm, but quail-chick chimera experiments revealed that the cranial neural crest (including the cardiac neural crest, a subset of the cranial neural crest that arises from the caudal hindbrain) provides perivascular cells to blood vessels in the face, pharyngeal arches and forebrain, including those of the retina (Le Lièvre and Le Douarin, 1975; Bergwerff et al., 1998; Etchevers et al., 2001; Korn et al., 2002). This was later supported via genetic lineage-tracing studies in mice (Jiang et al., 2000; Gage et al., 2005; Trost et al., 2013) and most recently zebrafish (Wang et al., 2014; Ando et al., 2016).

Multiple studies over the past decade, both *in vitro* and *in vivo*, have shown that Notch signalling is necessary for the formation of perivascular cells originating from both the mesoderm and the neural crest (e.g. Doi et al., 2006; Noseda et al., 2006; High et al., 2007, 2008; Liu et al., 2009, 2010; Chang et al., 2012; Manderfield et al., 2012, 2015; Wang et al., 2014; for reviews, see Gridley, 2007, 2010; Phng and Gerhardt, 2009; Boucher et al., 2012). Constitutive activation of the Notch pathway, via expression of the Notch1 intracellular domain (NICD), was sufficient to up-regulate *smooth muscle myosin heavy chain* (*Myh11*) and other smooth muscle marker genes in the C3H10T1/2 (mouse embryonic fibroblast) cell line (Doi et al., 2006). Physiological Notch activation, via co-culture with L cells stably expressing the Notch ligand Jagged1 (though not Delta-like 4), was also sufficient to up-regulate *Myh11* in this fibroblast cell line (Doi et al., 2006). In contrast, *NICD* transfection did not up-regulate *Myh11* in non-mesenchymal cell lines (mouse mammary gland epithelial cells, human umbilical vein endothelial cells, or human epidermal keratinocytes) (Doi et al., 2006). *In vivo*, NICD is sufficient to convert trunk mesoderm-derived somite cells to perivascular cells, at the expense of a muscle cell fate (Ben-Yair and Kalcheim, 2008; Mayeuf-Louchart et al., 2014). This was first demonstrated in chicken, by electroporating the lateral dermomyotome with *NICD* (Ben-Yair and Kalcheim, 2008), and more recently in mouse, by replacing one allele of the somite-expressed gene *Pax3* with *NICD* (Mayeuf-Louchart et al., 2014).

Here, we show that constitutively active Notch1 is also sufficient *in vivo* to drive a perivascular cell fate in cranial neural crest-derived frontonasal mesenchyme, and perhaps also in developing olfactory ensheathing glial cells (OECs). We originally aimed to test the effect of prematurely activating Notch1 on the development of OECs, which are derived from the cranial neural crest cells that colonise the frontonasal mass before the olfactory placode forms (Barraud et al., 2010). OECs are first detected on the chicken olfactory nerve at embryonic day (E)3.5, via immunoreactivity for the early glial marker myelin protein zero (Mpz, P0) (Drapkin and Silverman, 1999). Two days later, at E5.5, *Notch1* is up-regulated in developing OECs, and by E6.5, almost all developing OECs express Sox2 (Miller et al., 2016), which is a direct Notch/Rbpj target (Wakamatsu et al., 2004; Ehm et al., 2010; Li et al., 2012). In the development of Schwann cells, the glia of all other peripheral nerves, Notch signalling promotes the transition from Schwann cell precursors (which express Mpz) to immature Schwann cells (Woodhoo et al., 2009). To test the hypothesis that a similar Notch-mediated transition is important for OEC development, we aimed to activate Notch1 prematurely in developing chicken OECs, for which temporal control of the onset of Notch1 signalling would be required. Sato et al. (2008) previously used *in ovo* electroporation to insert into the genome of presomitic mesoderm cells both a construct that constitutively expresses the reverse (‘Tet-on’) tetracycline transactivator protein variant rtTA2^S^M2 (Urlinger et al., 2000), and a tetracycline-inducible *NotchΔE* construct, in which a single tetracycline-response element controls the bidirectional transcription of *Notch*Δ*E* (encoding a constitutively active extracellular deletion mutant of mouse Notch1; Kopan et al., 1996) and *EGFP*, whose expression was activated at somite stages by doxycycline injection. This resulted in the conversion of somite cells either to perivascular cells (also shown by electroporating a construct encoding *NICD* directly into the lateral dermomyotome; Ben-Yair and Kalcheim, 2008) or endothelial cells (Sato et al., 2008). Here, we used the conditional expression approach of Sato et al. (2008) to insert their tetracycline-inducible *NotchΔE/EGFP* construct into the genome of premigratory cranial neural crest cell precursors, and activate *NotchΔE/EGFP* expression from E4 (by doxycycline injection), 1.5 days before *Notch1* is normally up-regulated in developing OECs (Miller et al., 2016). To our surprise, we saw a striking phenotype in the neural crest-derived frontonasal mesenchyme (most of which would normally form skeletal or connective tissue, as well as perivascular cells), namely the formation by *NotchΔE/EGFP*-targeted cells of ectopic perivascular cells. *NotchΔE/EGFP*-targeted cells on the olfactory nerve also upregulated a perivascular marker. Hence, constitutive activation of Notch1 is sufficient *in vivo* to convert not only trunk mesoderm-derived somite cells (Ben-Yair and Kalcheim, 2008; Sato et al., 2008; Mayeuf-Louchart et al., 2014), but also cranial neural crest-derived frontonasal mesenchyme (and perhaps developing olfactory glia) to perivascular cells. These results suggest that during normal development, vascular endothelial cells expressing Notch ligands may recruit adjacent neural crest-derived frontonasal mesenchyme cells (and perhaps also developing olfactory glia) to form perivascular cells, via the sustained activation of Notch signalling. Furthermore, given that Notch signalling was not activated in targeted cranial neural crest-derived cells until after doxycycline was injected at E4, several days after the end of cranial neural crest migration, our data also speak to the plasticity of cranial neural crest-derived frontonasal mesenchyme and developing olfactory ensheathing glia.

## RESULTS

We used the Tol2 transposase/‘Tet-on’ *in ovo* electroporation system (Sato et al., 2007; Watanabe et al., 2007), which inserts tetracycline-dependent constructs into the genome of targeted cells, to drive constitutively active Notch1 expression in cranial neural crest-derived cells from embryonic day (E)4 [Hamburger–Hamilton (HH) stage 24; Hamburger and Hamilton, 1951]. Our original intention was to investigate the effect of premature Notch1 activation on the development of olfactory ensheathing cells (OECs, the glial cells of the olfactory nerve), which up-regulate *Notch1* from E5.5 (HH stage 24) (Miller et al., 2016). We therefore aimed to target the cranial neural crest precursors of OECs, which colonise the frontonasal mass before the olfactory placode forms (Barraud et al., 2010), with the Tol2-integratable, tetracycline-dependent construct *pT2K-Notch*Δ*E-BI-EGFP* (Sato et al., 2008). In this construct, a single tetracycline-response element controls the bidirectional transcription of *Notch*Δ*E* (encoding a constitutively active extracellular deletion mutant of mouse Notch1; Kopan et al., 1996) and *EGFP* (thus, EGFP labels cells successfully targeted with *Notch*Δ*E*; Sato et al., 2008).

We electroporated prospective cranial ectoderm *in ovo* at HH stages 6-8 (25-28 h of incubation) with *pT2K-Notch*Δ*E-BI-EGFP* (hereafter *Notch*Δ*E/EGFP*) or the Tol2-integratable control construct *pT2K-CAGGS-EGFP*, encoding EGFP only (Sato et al., 2007). Each of these constructs was co-electroporated with the Tol2-integratable construct *pT2K-CAGGS-rtTA2^S^M2* (Sato et al., 2007), encoding the reverse (‘Tet-on’) tetracycline transactivator protein rtTA2 under the control of the synthetic CAGGS promoter (Niwa et al., 1991) (thus providing a continuous supply of rtTA2 in targeted cells), plus the *pCAGGS-T2TP* construct, encoding Tol2 transposase (Sato et al., 2007) (to insert the *rtTA2* and *Notch*Δ*E/EGFP* or control *EGFP* constructs into the genome of targeted cells). Doxycycline was injected into the yolk under the embryo at E4 (HH stage 24) to initiate *Notch*Δ*E/EGFP* expression (the control *EGFP* is constitutively expressed). Embryos were collected 1-4 days later (E5-E8; HH stages 27-34) for sectioning, followed by *in situ* hybridisation plus immunohistochemistry on sections.

### Constitutive Notch activation from E4 converts frontonasal mesenchyme cells to perivascular cells

**Fig. 1.**
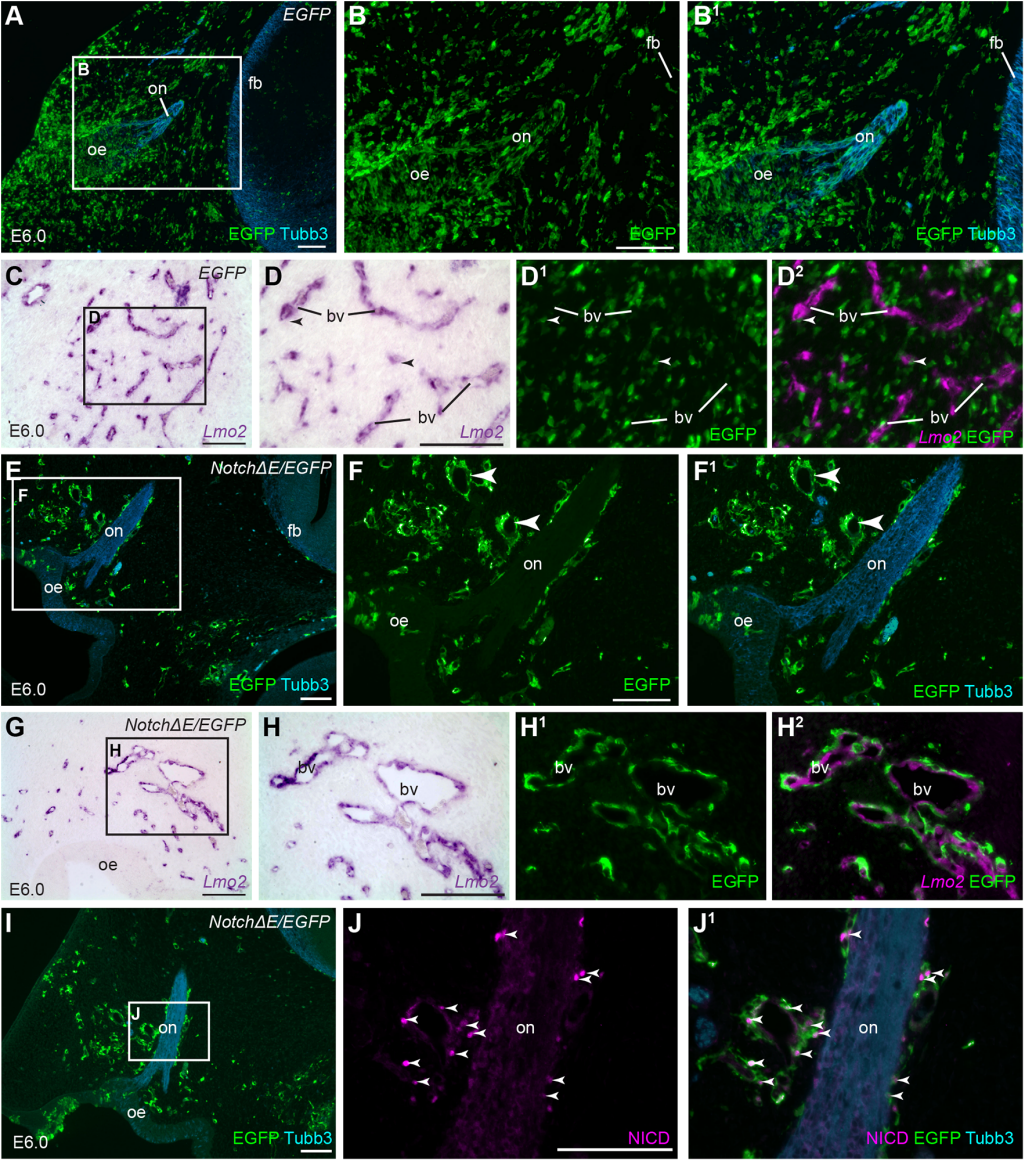
**Constitutive Notch activation from E4 causes frontonasal mesenchyme cells to associate with vascular endothelial cells.** Parasagittal sections of the olfactory region from chicken embryos in which the cranial ectoderm had been targeted *in ovo* at E1 with *EGFP* alone (control) or *NotchΔE*/*EGFP*, using the Tol2 transposase/‘Tet-on’ electroporation system (Sato et al., 2007; Watanabe et al., 2007). Eggs were injected with doxycycline at E4. (A-B^1^) A control *EGFP*-targeted embryo at E6. Immunostaining for EGFP and Tubb3 reveals *EGFP*-targeted cells throughout the mesenchyme and along the olfactory nerve, as well as in the forebrain, surface ectoderm and olfactory epithelium. (C-D^2^) In the same embryo at E6, *EGFP*-targeted cells are uniformly distributed in the frontonasal mesenchyme; only a few are associated with *Lmo2*-positive vascular endothelial cells (arrowheads in D-D^2^ highlight examples). (E-F^1^) A *NotchΔE*/*EGFP*-targeted embryo at E6. *NotchΔE*/*EGFP*-targeted cells aggregate in rings in the frontonasal mesenchyme (arrowheads in F,F^1^) and at the edges of the olfactory nerve. (G-H^2^) In the same embryo at E6, *NotchΔE*/*EGFP*-targeted cells encircle *Lmo2*-positive vascular endothelial cells. (I) The same *NotchΔE*/*EGFP*-targeted embryo at E6 shown in E-F^1^. Immunostaining for EGFP and Tubb3 shows rings of *NotchΔE*/*EGFP*-targeted cells in the frontonasal mesenchyme and along the edges of olfactory nerve. (J,J^1^) Higher-power view of the boxed region in I. Immunostaining for the cleaved Notch1 intracellular domain reveals co-localisation with *NotchΔE*/*EGFP*-targeted cells (arrowheads highlight examples, both in the mesenchyme and at the edges of the olfactory nerve). bv, blood vessel; EGFP, enhanced GFP; fb, forebrain; NICD, cleaved Notch1 intracellular domain; oe, olfactory epithelium; on, olfactory nerve. Scale bars: 100 µm.

At E6 (HH stage 29; two days after doxycycline injection) in control *EGFP*-targeted embryos (*n*=2), EGFP-positive cells are distributed throughout the frontonasal mesenchyme and along peripheral nerves (Fig. 1A-B^1^), with only a few EGFP-positive cells associated with *Lmo2*-positive vascular endothelium (Nagai and Sheng, 2008) (Fig. 1C-D^2^). In contrast, in *Notch*Δ*E/EGFP*-targeted embryos at E6-7 (HH stages 29-31; *n*=8), most EGFP-positive cells are aggregated in rings in the mesenchyme (Fig. 1E-F^1^), encircling *Lmo2*-positive vascular endothelium (Fig. 1G-H^2^). The same ‘ring-like’ distribution of EGFP-positive cells was also seen in *Notch*Δ*E/EGFP*-targeted embryos at E5 (HH stage 27; *n*=3) (not shown). Notch pathway activation in *Notch*Δ*E/EGFP*-targeted cells at E6 was confirmed by co-immunostaining for EGFP and the cleaved Notch1 intracellular domain (*n*=2; Fig. 1I-J^1^).

**Fig. 2.**
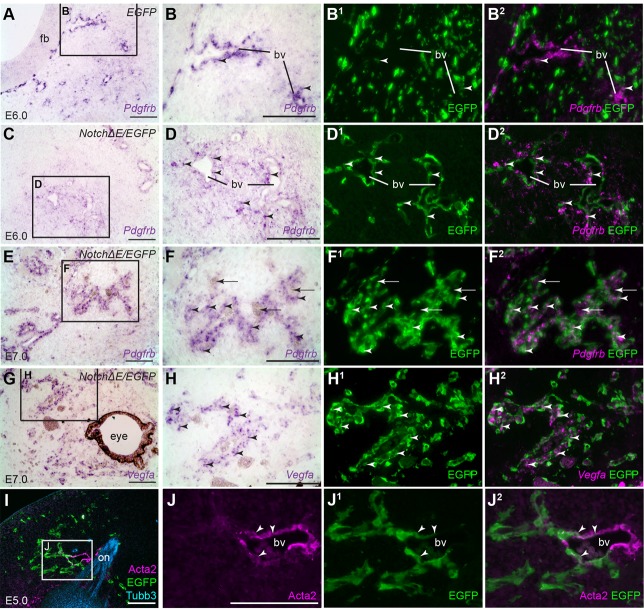
**Constitutive Notch activation from E4 converts frontonasal mesenchyme cells to perivascular cells.** Parasagittal sections of the frontonasal region from chicken embryos in which the cranial ectoderm had been targeted *in ovo* at E1 with *EGFP* alone (control) or *NotchΔE*/*EGFP*, using the Tol2 transposase/‘Tet-on’ electroporation system. Eggs were injected with doxycycline at E4. (A) A control *EGFP*-targeted embryo at E6. *In situ* hybridisation for *Pdgfrb* reveals perivascular cells in rings in the frontonasal mesenchyme and at the edge of the forebrain. (B) Higher-power view of boxed region in A, showing *Pdgfrb*-positive perivascular cells associated with a blood vessel near the forebrain. (B^1^,B^2^) Same section as B immunostained for EGFP, with *Pdgfrb* shown as a false-colour overlay in B^2^, reveals a fairly uniform distribution of *EGFP*-targeted cells in the mesenchyme and almost no co-localisation with *Pdgfrb,* apart from a few cells (arrowheads)*.* (C-D^2^) A *NotchΔE/EGFP*-targeted embryo at E6. *NotchΔE/EGFP*-targeted cells are found in rings around the developing blood vessels and express *Pdgfrb* (arrowheads highlight examples), showing they are perivascular cells. (E-F^2^) A *NotchΔE/EGFP*-targeted embryo at E7. Blood vessels in the frontonasal mesenchyme (arrows indicate blood cells) are surrounded by *NotchΔE/EGFP*-targeted, *Pdgfrb­*-positive perivascular cells (arrowheads). (G) In the same *NotchΔE/EGFP*-targeted embryo at E7, *Vegfa* is expressed in developing vasculature in the frontonasal mass. (H-H^2^) Higher-power view of the boxed region in G, revealing *Vegfa*-positive *NotchΔE/EGFP*-targeted cells (arrowheads). (I-J^2^) A *NotchΔE/EGFP*-targeted embryo at E5, with *NotchΔE/EGFP*-targeted cells in rings in the frontonasal mesenchyme. Immunostaining for alpha-smooth muscle actin (Acta2) reveals a few Acta2-positive *NotchΔE/EGFP*-targeted cells (arrowheads) associated with a large blood vessel near the olfactory nerve. bv, blood vessel; EGFP, enhanced GFP; fb, forebrain; on, olfactory nerve. Scale bars: 100 µm.

Since cranial neural crest cells normally give rise to perivascular cells in the blood vessels of the face and forebrain (Etchevers et al., 2001), we wished to use molecular markers to test whether the *Notch*Δ*E/EGFP*-targeted cells encircling *Lmo2*-positive vascular endothelium in the frontonasal mesenchyme were indeed adopting a perivascular cell fate. There are no exclusive molecular markers for perivascular cells; furthermore, the expression levels of the various markers used can vary, depending on, for example, the developmental state of the cells (reviewed by Armulik et al., 2011). Nevertheless, one commonly used perivascular cell marker is *platelet-derived growth factor receptor beta* (*Pdgfrb*) (reviewed by Armulik et al., 2011). After doxycycline injection at E4, control *EGFP*-targeted embryos at E6 show almost no co-localisation between EGFP and *Pdgfrb* (*n*=3), barring a few cells associated with the vasculature, as expected (Fig. 2A-B^2^). In contrast, most *Notch*Δ*E/EGFP*-targeted cells in the frontonasal mesenchyme express *Pdgfrb* at E6-7 (*n*=5) (Fig. 2C-D^2^). Perivascular cells also express *vascular endothelial growth factor A* (*Vegfa*) (Darland et al., 2003; Parenti et al., 2004; Kale et al., 2005). After initiating constitutive Notch activity by injecting doxycycline at E4, we detected *Vegfa* expression in *Notch*Δ*E/EGFP*-targeted cells at E6-E7 (*n*=2; Fig. 2G-H^2^). Furthermore, immunoreactivity for the smooth muscle/myofibroblast marker alpha-smooth muscle actin (Acta2; reviewed by Armulik et al., 2011) was detected in some *Notch*Δ*E/EGFP*-targeted cells associated with larger blood vessels at E5-E8 (*n*=2; Fig. 2I-J^2^).

Overall, these data suggest that constitutive Notch activation from E4 in cranial neural crest-derived frontonasal mesenchyme cells is sufficient to convert them to perivascular cells, identified by their location (i.e. encircling vascular endothelial cells in developing blood vessels) in combination with the expression of characteristic perivascular cell markers.

### Constitutive Notch activation from E4 may convert developing olfactory ensheathing cells into perivascular cells

**Fig. 3.**
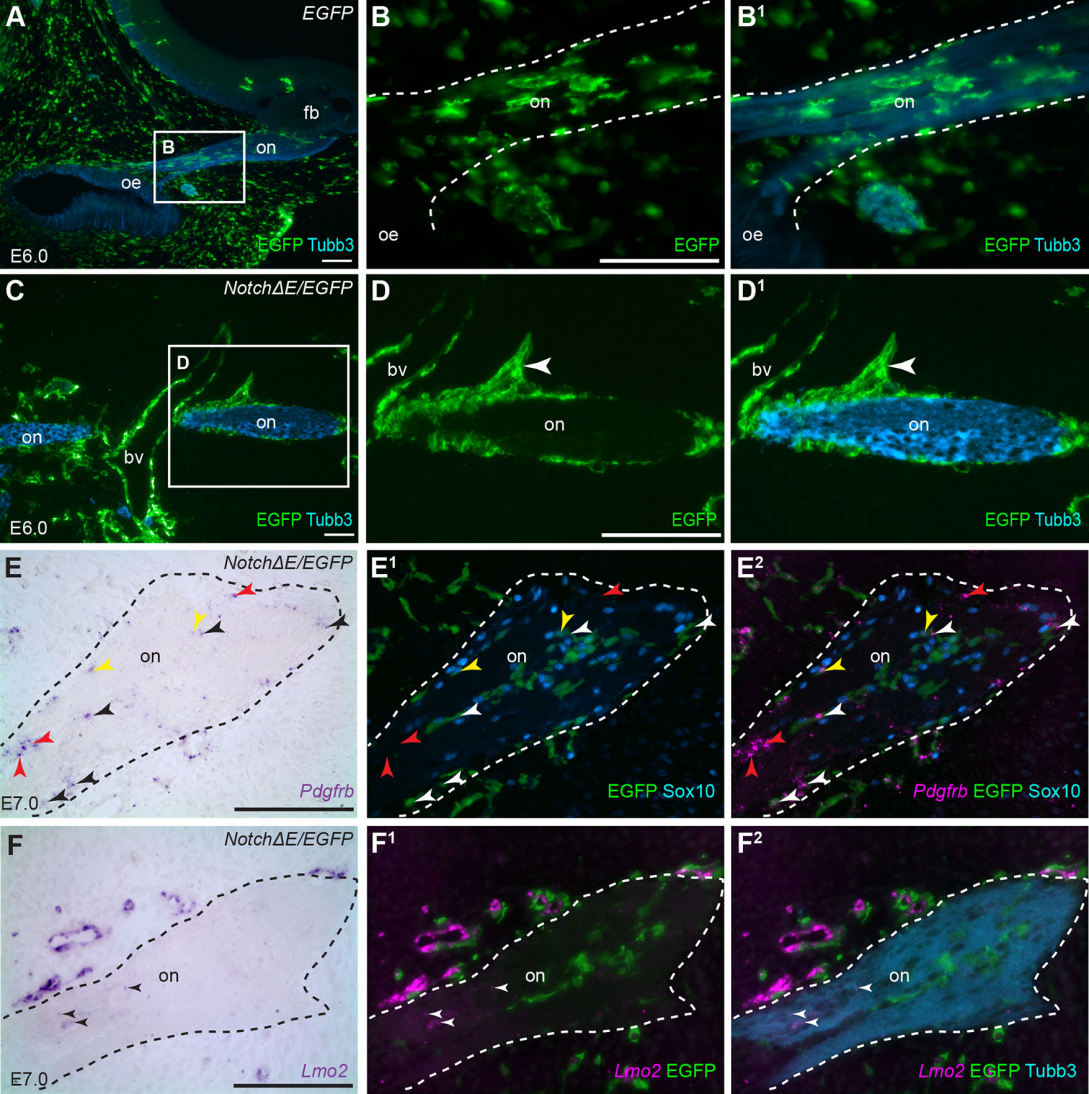
**Constitutive Notch activation from E4 converts developing olfactory ensheathing cells into perivascular cells.** Parasagittal sections of the olfactory region from chicken embryos in which the cranial ectoderm had been targeted *in ovo* at E1 with *EGFP* alone (control) or *NotchΔE*/*EGFP*, using the Tol2 transposase/‘Tet-on’ electroporation system. Eggs were injected with doxycycline at E4. Dotted lines demarcate the olfactory nerve. (A-B^1^) A control *EGFP*-targeted embryo at E6, in which the olfactory placode was not targeted. *EGFP*-targeted neural crest-derived cells are found throughout the frontonasal mesenchyme and associated with Tubb3-positive olfactory axons, presumably developing OECs. (C-D^1^) A *NotchΔE*/*EGFP*-targeted embryo at E6. *NotchΔE*/*EGFP*-targeted cells associated with the olfactory nerve are aggregated at the edges of the nerve, rather than being found throughout the nerve, and form processes extending away from it (arrowhead in D,D^1^). (E-E^2^) In a *NotchΔE*/*EGFP*-targeted embryo at E7, *in situ* hybridisation for the perivascular marker *Pdgfrb* (shown as a false-colour overlay in E^1^,E^2^), combined with immunostaining for the OEC marker Sox10, shows that some *NotchΔE*/*EGFP*-targeted cells are developing OECs; a few of these co-express *Pdgfrb* (yellow arrowheads), suggesting they may be undergoing fate conversion. Some *NotchΔE*/*EGFP*-targeted cells express *Pdgfrb* but not Sox10 (black/white arrowheads). Some untargeted cells express *Pdgfrb* (red arrowheads). (F-F^2^) In a nearby section from the same *NotchΔE*/*EGFP*-targeted embryo at E7, *in situ* hybridisation for *Lmo2* reveals a few weakly *Lmo2*-positive vascular endothelial cells on the olfactory nerve (arrowheads). bv, blood vessel; EGFP, enhanced GFP; fb, forebrain; oe, olfactory epithelium; on, olfactory nerve; pn, peripheral nerve. Scale bars: 100 μm.

In control *EGFP*-targeted embryos at E6 (two days after doxycycline injection), EGFP-positive developing OECs (which are neural crest-derived; Barraud et al., 2010) are distributed throughout the olfactory nerve, among the axons (Fig. 3A-B^1^). In contrast, in *Notch*Δ*E/EGFP*-targeted embryos at E6 (*n*=4), most *Notch*Δ*E/EGFP*-targeted cells on the olfactory nerve seem to be excluded from the nerve's interior, instead aggregating at the edges of the nerve in ‘processes’ extending away from it (Fig. 3C-D^1^). At least some *Notch*Δ*E/EGFP*-targeted cells on the olfactory nerve at E6-7 express the perivascular cell marker *Pdgfrb* (*n*=2; Fig. 3E-E^2^), suggesting that, like *Notch*Δ*E/EGFP*-targeted cells in the frontonasal mesenchyme, they may have been converted to perivascular cells. Several of the *Notch*Δ*E/EGFP*-targeted cells on the olfactory nerve express the OEC marker Sox10 (Barraud et al., 2010) (Fig. 3E-E^2^), confirming that at least some developing OECs were targeted. Indeed, a few of the *Notch*Δ*E/EGFP*-targeted cells co-express Sox10 and *Pdgfrb* (yellow arrowheads, Fig. 3E-E^2^), suggesting they may have been caught in the process of changing fate. Some of the *Notch*Δ*E/EGFP*-targeted cells on the olfactory nerve are *Pdgfrb*-positive but Sox10-negative (black/white arrowheads, Fig. 3E-E^2^): these may have originated from *Notch*Δ*E/EGFP*-targeted developing OECs that have already down-regulated Sox10 expression, or *Notch*Δ*E/EGFP*-targeted frontonasal mesenchyme cells that have colonised the nerve. The endogenous olfactory nerve microvasculature is starting to form at this time; *in situ* hybridisation for *Pdgfrb* and the vascular endothelial cell marker *Lmo2* on sections of both *Notch*Δ*E/EGFP*-targeted and wild-type embryos at E6.5-7 (*n*=3) reveals some untargeted *Pdgfrb*-positive cells (red arrowheads, Fig. 3E-E^2^) and a few *Lmo2*-positive cells (Fig. 3F-F^2^) within the olfactory nerve.

### Vasculature containing *Notch*Δ*E/EGFP*-targeted perivascular cells seems to attract peripheral axons and glia

**Fig. 4.**
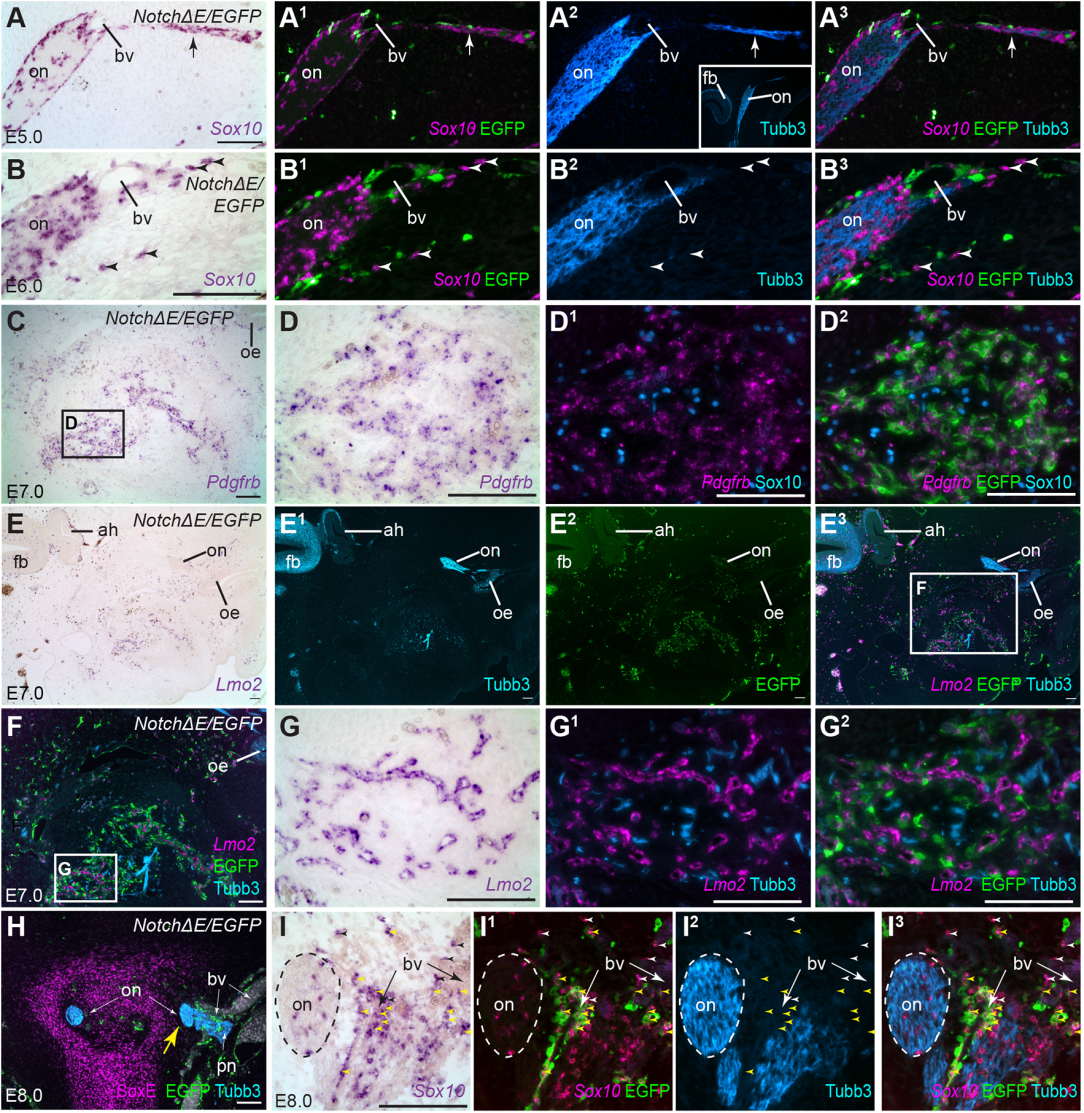
**Peripheral axons and glia seem to be attracted to blood vessels containing *NotchΔE*/*EGFP*-targeted cells.** Parasagittal (A-G^2^) and coronal (H-I^3^) sections from embryos in which the cranial ectoderm had been targeted *in ovo* at E1 with *NotchΔE*/*EGFP*, using the Tol2 transposase/‘Tet-on’ electroporation system. Eggs were injected with doxycycline at E4. (A) In an E5 embryo, *in situ* hybridisation for *Sox10* reveals developing OECs on the olfactory nerve. (A^1^-A^3^) Same section as A, immunostained for EGFP and Tubb3, with *Sox10* shown as a false-colour overlay in A^2^,A^3^. A thin nerve branch (arrow) deviates from the olfactory nerve away from the forebrain (for orientation, see low-power inset in A^2^). The branch-point is near a developing blood vessel, whose wall contains *NotchΔE*/*EGFP*-targeted cells. (B-B^3^) In an E6 embryo, several untargeted *Sox10*-positive cells (arrowheads), presumably developing OECs, are found isolated in the mesenchyme at some distance from the olfactory nerve, near *NotchΔE*/*EGFP*-targeted cells. (C-D^2^) In an E7 embryo, *in situ* hybridisation for *Pdgfrb* followed by immunostaining for EGFP and Sox10 reveals that many *NotchΔE*/*EGFP*-targeted cells have formed *Pdgfrb*-positive perivascular cells, with which many Sox10-positive cells (presumably peripheral glial cells) are associated. This is far from the olfactory nerve: note the location of the olfactory epithelium at the top right. (E-G^2^) A nearby section of the same E7 embryo, shown at low-power in E-E^3^ for orientation (note the position of the olfactory epithelium and olfactory nerve towards the top right, and the forebrain and adenohypophysis towards the top left). *In situ* hybridisation for *Lmo2* and immunostaining for EGFP and Tubb3 confirm the presence of peripheral axons (and possibly neurons) close to a large concentration of *NotchΔE*/*EGFP*-targeted cells that are associated with *Lmo2*-positive vascular endothelium. (H) In an E8 embryo (coronal section), the entire olfactory nerve on one side is misplaced laterally (yellow arrow) towards several large blood vessels whose walls contain *NotchΔE*/*EGFP*-targeted cells. The displaced olfactory nerve is in contact with another peripheral nerve, and no longer surrounded by cartilage (identified by immunostaining with an anti-Sox9 antibody that also cross-reacts with other SoxE family members), unlike the olfactory nerve on the other side. (I-I^3^) In a nearby section of the same E8 embryo, *in situ* hybridisation for *Sox10* and immunostaining for EGFP and Tubb3 show that some *Sox10*-positive OECs – both untargeted (black/white arrowheads) and *NotchΔE*/*EGFP*-targeted (yellow arrowheads) – are found at a distance from axons, associated instead with blood vessels whose walls contain *NotchΔE*/*EGFP*-targeted cells. ah, adenohypophysis; bv, blood vessel; EGFP, enhanced GFP; fb, forebrain; oe, olfactory epithelium; on, olfactory nerve; pn, peripheral nerve. Scale bars: 100 µm.

In half of the *Notch*Δ*E/EGFP*-targeted embryos at E5-8 (*n*=6 out of 12), olfactory and other peripheral axons and their accompanying OECs/Schwann cells seemed to project towards vasculature containing *Notch*Δ*E/EGFP*-targeted cells, with some of the glial cells (identified by Sox10 expression; Barraud et al., 2010; Jacob, 2015) even found isolated from axons, in association with such cells. Fig. 4A-A^3^ shows an example at E5, in which the olfactory nerve is in contact with such a blood vessel, at which point olfactory axons seem to project in the wrong direction, away from the forebrain. Fig. 4B-B^3^ shows an example at E6, in which the olfactory nerve is in close contact with vasculature containing *Notch*Δ*E/EGFP*-targeted cells, towards which untargeted *Sox10*-positive OECs seem to have migrated, leaving the olfactory nerve altogether. Fig. 4C-G^2^ shows another example at E7, in which *Notch*Δ*E/EGFP*-targeted, *Pdgfrb*-positive perivascular cells are closely associated with Sox10-positive glial cells (Fig. 4D^1^,D^2^) and axons (and possibly neurons) caudal to the olfactory system (Fig. 4G^1^,G^2^). In one *Notch*Δ*E/EGFP*-targeted embryo at E8 (HH stage 34), the entire olfactory nerve on one side is misplaced laterally, outside the cartilage that normally encloses it, apparently projecting towards large blood vessels containing *Notch*Δ*E/EGFP*-targeted cells (Fig. 4H). On a nearby section from the same embryo, many *Sox10*-positive OECs (both *Notch*Δ*E/EGFP*-targeted and untargeted) seem to have migrated away from the olfactory nerve altogether, instead associating with blood vessels containing *Notch*Δ*E/EGFP*-targeted cells (Fig. 4I-I^3^). Taken together, these results suggest that vasculature containing *Notch*Δ*E/EGFP*-targeted cells attracts both peripheral axons and glia.

## DISCUSSION

In experiments originally aimed at testing the effect on olfactory ensheathing cell (OEC) development of prematurely activating Notch1, which is normally expressed in developing chicken OECs from E5 (Miller et al., 2016), we used the Tol2 transposase/‘Tet-on’ *in ovo* electroporation system (Sato et al., 2007; Watanabe et al., 2007) to drive *Notch*Δ*E*, encoding a constitutively active form of mouse Notch1 (Kopan et al., 1996; Sato et al., 2008), in cranial neural crest-derived cells from E4. This proved to be sufficient to convert both frontonasal mesenchyme cells, and perhaps also developing OECs, to *Pdgfrb*-positive perivascular cells. *Pdgfrb* encodes a receptor tyrosine kinase required in pericytes during angiogenesis, for their recruitment to sprouting capillaries and proliferation (Lindahl et al., 1997; Hellström et al., 1999; Winkler et al., 2010). In the frontonasal mesenchyme at E5-7, ectopic *Notch*Δ*E/EGFP*-targeted perivascular cells were found encircling *Lmo2*-positive vascular endothelium. *Vegfr2* (*Flk1*, *Kdr*)-expressing angioblasts are found throughout the developing cranial mesenchyme in both chicken and mouse (Couly et al., 1995; Yoshida et al., 2008); in chicken, these initially dispersed *Vegfr2*-positive cells have all incorporated into blood vessels by E3-4 (Couly et al., 1995). Hence, expression of constitutively active Notch1 from E4 in cranial neural crest-derived frontonasal mesenchyme cells causes them to adopt a perivascular cell fate and associate with the vascular endothelium of nearby blood vessels.

*Notch*Δ*E/EGFP*-targeted *Pdgfrb*-positive cells were also seen within the olfactory nerve, suggesting that constitutive Notch1 activation from E4 within developing OECs (which can first be identified at E3.5, by myelin protein zero immunoreactivity; Drapkin and Silverman, 1999) could be sufficient to convert them to a perivascular cell fate. Indeed, some of the *Notch*Δ*E/EGFP*-targeted, *Pdgfrb*-positive cells on the olfactory nerve co-expressed the OEC marker Sox10 (Barraud et al., 2010), suggesting they were in the process of changing fate. Most *Notch*Δ*E/EGFP*-targeted cells seemed to be excluded from the interior of the olfactory nerve and instead aggregated together at the edges, projecting away from the nerve. This may reflect the lack of blood vessels inside developing nerves until relatively late in development, given that we did not see many *Lmo2*-positive vascular endothelial cells inside the chicken olfactory nerve at E6.5-7 (in the rat sciatic nerve, blood vessels are first seen only at E18; Wanner et al., 2006). The presence of some untargeted *Pdgfrb*-positive cells within the olfactory nerve at E6.5-7 also suggests that perivascular cells are normally beginning to differentiate at this stage. Taken together, these data may also suggest that at least some of the perivascular cells of the olfactory nerve vasculature derive from developing OECs, in response to sustained Notch1 activation. This is in contrast to the trunk, where only endoneurial fibroblasts, and not endoneurial perivascular cells, derive from Schwann cell precursors (Joseph et al., 2004). Furthermore, since expression of the constitutively active Notch1 mutant protein was only activated in targeted cranial neural crest-derived cells following doxycycline injection at E4, our findings also reveal the plasticity of cranial neural crest-derived frontonasal mesenchyme and developing olfactory ensheathing glia.

Our results are consistent with previous work showing that constitutive Notch1 activation (via expression of the Notch1 intracellular domain) in trunk mesoderm-derived somite cells promotes adoption of a perivascular fate at the expense of a skeletal muscle fate (Ben-Yair and Kalcheim, 2008; Sato et al., 2008; Mayeuf-Louchart et al., 2014); they also extend this finding to cranial neural crest-derived cells. The Notch pathway plays critical roles in many aspects of vascular development, including perivascular cell recruitment and differentiation during vasculogenesis (i.e. the formation of new blood vessels *de novo*) in addition to maturation, stabilization and remodelling of the vasculature during angiogenesis (i.e. the formation of new blood vessels by sprouting from existing vessels) (reviewed by Gridley, 2007; Phng and Gerhardt, 2009; Gridley, 2010; Boucher et al., 2012). Our data suggest that constitutive Notch1 signalling from E4 in cranial neural crest-derived frontonasal mesenchyme and developing OECs promotes a perivascular cell fate. Since Notch signalling is required for neural crest-derived perivascular cell formation (High et al., 2007, 2008; Chang et al., 2012; Manderfield et al., 2012; Wang et al., 2014; Manderfield et al., 2015), this likely reflects a normal developmental process, whereby vascular endothelial cells expressing Notch ligands recruit adjacent frontonasal mesenchyme cells to form perivascular cells through sustained activation of Notch signalling.

Consistent with this hypothesis, sustained activation of Notch signalling [via exposure to Delta-like 4 (Dll4) from endothelial cells] is both sufficient and necessary for conversion of skeletal myoblasts to pericytes *in vitro*: silencing of Dll4 restores myogenesis (Cappellari et al., 2013). *In vivo*, expression of the Notch1 intracellular domain in MyoD-positive muscle cells also drives a pericyte fate, while occasional perivascular cells in wild-type embryos are derived from Myf5- or MyoD-expressing precursors (Cappellari et al., 2013). This suggests that Notch ligand production from vascular endothelium in skeletal muscle may sometimes induce a fate switch in adjacent myoblasts. Sustained Notch signalling is also required in vascular smooth muscle cells to suppress alternative fates and maintain the perivascular fate: in the absence of the Notch ligand Jagged1, mouse somite-derived vascular smooth muscle cells adopt a chondrocyte fate, which can lead to vessel ossification (Briot et al., 2014). Thus, sustained Notch signalling appears not only to promote, but also maintain, the perivascular cell fate.

We also found that vasculature containing *Notch*Δ*E/EGFP*-targeted perivascular cells seemed to attract peripheral axons and their associated glia (OECs on the olfactory nerve; Schwann cells on all other nerves), with some Sox10-positive glial cells appearing to have left the nerve altogether. We identified *Vegfa* expression in *Notch*Δ*E/EGFP*-targeted perivascular cells. *Vegfa* is expressed by pericytes in the developing retinal vasculature (where pericytes are neural crest-derived; Etchevers et al., 2001; Trost et al., 2013); in heterozygous *Vegfa^lacZ^* transgenic mice (in which *lacZ* under an independent ribosome entry site was inserted into the 3′ untranslated region of the *Vegfa* gene; Miquerol et al., 1999), retinal pericytes express beta-galactosidase (Darland et al., 2003). Vegfa is also secreted by perivascular cells induced from 10T1/2 cells by co-culturing with endothelial cells (Darland et al., 2003). Vegfa is not only a pro-angiogenic factor (reviewed by Jin et al., 2014; Moens et al., 2014) but is also secreted by Schwann cells, acting in an autocrine loop to enhance Schwann cell proliferation and migration, and also promoting axon outgrowth via Vegfr2 (reviewed by Rosenstein et al., 2010). Thus it is possible that Vegfa secreted by *Notch*Δ*E/EGFP*-targeted perivascular cells attracts OECs/Schwann cells, and at least in some cases olfactory axons, towards the vasculature.

Overall, our data support and extend previous work showing that the Notch pathway is necessary for the formation of perivascular cells from the cranial neural crest (High et al., 2007, 2008; Chang et al., 2012; Manderfield et al., 2012; Wang et al., 2014; Manderfield et al., 2015), by showing that constitutively active Notch1 promotes a perivascular cell fate in frontonasal mesenchyme, and perhaps also in glial progenitors on the olfactory nerve, several days after the end of cranial neural crest migration. Intriguingly, constitutive activation of Notch signalling via expression of the Notch3 intracellular domain seems to promote the proliferation, but not the specification, of brain pericytes in zebrafish (Wang et al., 2014), suggesting that the activation of distinct Notch signalling pathways may have different outcomes during the development of perivascular cells.

## MATERIALS AND METHODS

### Electroporation constructs

All electroporation constructs were kind gifts of Yoshiko Takahashi (Kyoto University, Kyoto, Japan); the *pT2K-Notch*Δ*E-BI-EGFP* construct (Sato et al., 2008) was used with the kind permission of Raphael Kopan (Washington University, St Louis, MO, USA). Constructs were prepared using the EndoFree Plasmid Maxi kit (Qiagen) to a stock concentration of 5 μg/μl. *pCAGGS-T2TP* (Kawakami and Noda, 2004; Sato et al., 2007) encodes Tol2 transposase under the control of the synthetic CAGGS promoter (Niwa et al., 1991); the Tol2-integratable *pT2K-CAGGS-rtTA2^S^M2* construct (Sato et al., 2007) encodes the reverse (‘Tet-on’) tetracycline transactivator protein variant rtTA2^S^M2 (Urlinger et al., 2000); the Tol2-integratable, tetracycline-dependent *pT2K-Notch*Δ*E-BI-EGFP* construct (Sato et al., 2008) encodes a constitutively active extracellular deletion mutant of mouse Notch1 (*Notch*Δ*E*; Kopan et al., 1996) and *EGFP*, bidirectionally transcribed under the control of a single tetracycline-response element; the Tol2-integratable *pT2K-CAGGS-EGFP* control construct (Sato et al., 2007) encodes EGFP alone.

### *In ovo* electroporation

Fertilised chicken (*Gallus gallus domesticus*) eggs were obtained from commercial sources. All work with chicken embryos was conducted in accordance with the UK Animals (Scientific Procedures) Act 1986. Eggs were incubated in a humidified atmosphere at 38°C for 25-28 h to reach Hamburger–Hamilton stages 6-8 (Hamburger and Hamilton, 1951) (between the head-fold stage and the 4-somite stage). Black ink (Fount India, Pelikan) was diluted to 1% in filtered phosphate-buffered saline (PBS) and injected underneath the blastoderm to visualise the embryo. The cranial ectoderm and neural folds were co-electroporated with 1:1:1 *pCAGGS-T2TP*, *pT2K-CAGGS-rtTA2^S^M2* and either *pT2K-Notch*Δ*E-BI-EGFP* or control *pT2K-CAGGS-EGFP*, to a final concentration of 0.9 μg/μl each, mixed with Fast Green to a final dilution of 2% and sucrose to a final concentration of 8%. The positive electrode was placed in the yolk underneath the head process and perpendicular to the cranial–caudal axis of the embryo. The plasmid solution was micro-pipetted over the cranial ectoderm and the negative ‘spoon-type’ electrode brought down over the embryo, as described (Brown et al., 2012). An ECM 830 Square Wave Pulse generator (BTX Instrument Division, Harvard Apparatus, Inc.) was used to apply five 50-ms 5 V pulses at 100 ms intervals. The egg was sealed with Parafilm and returned to the incubator. At embryonic day (E)4, 500 μl of doxycycline solution (100 μg/μl doxycycline in water) was injected under the embryo. The egg was re-sealed and returned to the incubator until the desired stage. Surviving embryos were fixed in modified Carnoy's (6 volumes ethanol, 3 volumes 37% formaldehyde, 1 volume glacial acetic acid), dehydrated into ethanol, cleared in Histosol (National Diagnostics) and embedded in paraffin wax for sectioning at 6 μm on a rotary microtome (Microm).

### Riboprobes

Chicken *Lmo2* (Nakazawa et al., 2006) was a kind gift of Guojun Sheng (RIKEN Center for Developmental Biology, Kobe, Japan). Chicken *Sox10* (Cheng et al., 2000) was a kind gift of Marianne Bronner (Caltech, Pasadena, CA, USA). An 803-bp fragment of chicken *Pdgfrb* cDNA, corresponding to base-pairs 1486-2288 (NCBI reference sequence XM_001233829.3) was PCR-amplified from cDNA (forward primer TAACGTGCTCTGCTGAAGGG; reverse primer CAGGAAGGTGTGCTTGTTGC), cloned into pDrive (Qiagen) using the Qiagen PCR cloning kit and sequenced (Biochemistry Department DNA Sequencing Facility, Cambridge, UK). A 608-bp fragment of chicken *Vegfa* cDNA, corresponding to base-pairs 631-1238 (NCBI reference sequence NM_205042.2) was similarly PCR-amplified (forward primer GCGGAAGCCCAACGAAGTTA; reverse primer TGTCCAGGCGAGAAATCAGG) and cloned into pDrive. PCR primers were designed and specificity checked using Primer-BLAST software from NCBI (Ye et al., 2012). The OligoCalc program (Kibbe, 2007) (http://www.basic.northwestern.edu/biotools/oligocalc.html) was used to check primer melting temperature and self-complementarity. Single-strand cDNA was prepared using Invitrogen's Superscript III First-Strand Synthesis System kit on total RNA extracted with Trizol (Invitrogen) from chicken embryos at E3.5 and E6.5. Digoxigenin-labelled antisense riboprobes were generated as described (Henrique et al., 1995).

### *In situ* hybridisation on sections

Slides were de-waxed in Histosol (National Diagnostics) and rehydrated through a graded ethanol series into diethylpyrocarbonate (DEPC)-treated PBS. Digoxigenin-labelled antisense riboprobes diluted 1:250 to 1:1000 in hybridisation buffer [1x salt solution (0.2 M NaCl, 10 mM Tris pH 7.5, 5 mM NaH_2_PO_4_.H_2_O, 5 mM Na_2_HPO_4_, 5 mM EDTA), 50% formamide, 10% dextran sulfate, 1 mg/ml yeast tRNA, 1x Denhardt's solution] were hybridised to sections overnight at 68°C. Slides were washed three times in wash solution (50% formamide, 1× SSC, 0.1% Tween-20) for 30 min to one hour each at 70°C, then given two 10-min washes in MABT (1x maleic acid buffer with 0.1% Tween-20) (10× MAB: 1 M maleic acid, 1.5 M NaCl, pH 7.5) at room temperature. Slides were incubated for at least 2 h in blocking solution [1% blocking reagent (Roche), 20% heat-denatured normal sheep serum (Sigma) in MABT]. Alkaline phosphatase-conjugated anti-digoxigenin antibody (Roche) was diluted 1:1500 in blocking solution and slides were incubated in the antibody solution overnight at room temperature. After five 30-min washes in MABT, slides were equilibrated via two 10-min washes in NTMT (0.1 M NaCl, 0.1 M Tris, pH 9.5, 50 mM MgCl_2_, 0.1% Tween-20), and the colour reaction performed in 20 μl/ml NBT/BCIP (Roche) in NTMT. Once the colour had developed to the desired extent, sections were washed twice in distilled water and once in PBS, then fixed for 5 min in 4% formaldehyde (Thermo Scientific) in PBS.

### Immunohistochemistry

Whether after fixation following *in situ* hybridisation as described in the preceding section, or after de-waxing and rehydrating untreated slides as described in the preceding section, slides were rinsed in PBS, blocked for 1 h at room temperature in 10% sheep serum in PBS with 0.1% Triton X-100 and then incubated overnight at 4°C with primary antibodies in blocking solution. (When the antibody against cleaved Notch1 intracellular domain was used, antigen retrieval was performed prior to blocking, by heating the slides for 4 min until boiling in a microwave in 10 mM sodium citrate buffer solution, pH 6, followed by two washes in PBS.) After three 5-10 min washes in PBS, appropriately matched Alexa Fluor-conjugated secondary antibodies (Molecular Probes) were applied at 1:1000 in the same blocking solution and incubated at room temperature for 2-3 h. If three primary antibodies were used, a biotinylated (instead of Alexa Fluor-conjugated) secondary antibody was used against Tubb3 (1:50 goat anti-mouse IgG2a, Invitrogen, or 1:250 horse anti-mouse IgG, Vector Laboratories), and, after three 5-10 min washes in PBS, the slides were further incubated for 1-2 h at room temperature with Alexa Fluor 350-conjugated NeutrAvidin (Molecular Probes) diluted 1:100 in filtered PBS. After three 5-10 min washes in PBS, slides were mounted in Fluoromount G (Southern Biotech). Primary antibodies used were: anti-Acta2 (mouse IgG2a, Sigma-Aldrich A5228, 1:500); anti-EGFP (rabbit, Invitrogen A-6455, 1:500; mouse IgG1, Roche 1814460001, 1:500); anti-activated Notch1 (cleaved Notch1 intracellular domain) (rabbit, Abcam ab8925, 1:150); anti-Sox10 (Meng et al., 2011; Yardley and García-Castro, 2012) (rabbit, kind gift of Vivian Lee, Medical College of Wisconsin, WI, USA, 1:3000); anti-Tubb3 (neuronal class III beta-tubulin) (clone TUJ1, mouse IgG2a, Covance MMS-435P, 1:500).

### Image capture and processing

Images were captured on a Zeiss AxioSkop 2 MOT compound microscope using QCapture Pro 6.0 software, a QImaging Retiga 2000R camera and an RGB pancake (QImaging), and processed using Adobe Photoshop CS6. To show co-localisation, bright-field *in situ* hybridisation images were inverted and inserted into the green channel only, then used as a false-colour overlay with immunofluorescence images of the same section.
